# Targeting ESM1/ VEGFα signaling axis: a promising therapeutic avenue for angiogenesis in cervical squamous cell carcinoma

**DOI:** 10.7150/jca.84654

**Published:** 2023-06-12

**Authors:** Dan Li, Xiaomin Su, Shen Xue, Li Yao, Dan Yu, Xianbing Tang, Yugang Huang

**Affiliations:** 1 Department of Pathology, Taihe Hospital, Hubei University of Medicine, Shiyan 442000, China.; 2 Department of Immunology, Nankai University School of Medicine, Tianjin 300110, China.; 3 Department of obstetrics and gynecology, Sinopharm Dongfeng General Hospital, Hubei University of Medicine, Shiyan 442008, China.

**Keywords:** ESM1, cervical squamous cell carcinoma, carcinoma angiogenesis, VEGFα

## Abstract

**Background:** Endothelial-specific molecule 1 (ESM1) dysregulation is widespread in various malignancies. However, the exact significance of ESM1 in cervical squamous cell carcinoma (CSCC) is not yet well understood.

**Methods:** The expression of ESM1 in CSCC was probed by immunohistochemistry (IHC) assay using human specimens and validated and explored ESM1 in CSCC based on TNMplot and TCGA (The Cancer Genome Atlas Program) data repository. Further, the GSEA analysis and *in vitro* experiments of human CSCC cell lines, including SiHa and ME-180, were performed to investigate the masked molecular mechanisms of ESM1 in CSCC.

**Results:** ESM1 was overexpressed in clinical CSCC tissues compared with paracancer controls, was an independent prognostic factor and was associated with poor prognosis in CSCC patients. These findings were further confirmed in the TNMplot and TCGA datasets. Furthermore, GSEA analysis revealed that the ESM1 high expression group was significantly enriched in carcinoma angiogenesis and the VEGFα signaling pathway. In addition, *in vitro* assays with human CSCC cell lines, including SiHa and ME-180, demonstrated that knockdown of ESM1 expression inhibited tumor cell proliferation, migration and invasion, resulting in attenuated VEGFα expression and blocked phosphorylation of VEGFR2 and ERK-1/2.

**Conclusion:** In CSCC patients, ESM1 was considerably overexpressed. Upregulation of ESM1 is predictive of poor clinical outcomes in CSCC. Furthermore, ESM1 overexpression promoted carcinoma angiogenesis and CSCC progression through the VEGF/ERK signaling pathway. Hence, ESM1 and associated genes might be useful prognostic biomarkers or therapeutic targets for CSCC individuals.

## Introduction

Squamous cell carcinoma of the cervix (CSCC) is a common reproductive malignancy with a significant incidence and fatality risk, contributing to around 15% of all cancer-related deaths in women worldwide^1^. Invasive squamous cell carcinoma, the most frequent pathological form, accounts for 75%-80% of cervical cancer^2^. The vast majority of CSCC is induced by the persistence of infection with high-risk human papillomavirus (HR-HPV); the availability of HPV vaccines, especially the 9-valent HPV vaccine, has yielded tremendous dividends for the prophylaxis of cervical cancer^3^, ^4^. However, the mortality rate of cervical cancer has not diminished markedly in the last few decades^5^. The gradually accumulating literature suggests that the occurrence and development of CSCC is a complex pathological event induced by a variety of predisposing factors and signaling pathways^6^-^8^.

Studies on molecular and genetic abnormalities predisposing to CSCC carcinogenesis have evolved considerably over the past few decades, providing insight into the molecular processes of cervical cancer and guiding the invention of novel therapeutic agents. Somatic mutations, including KRAS^9^, TP53^10^, PTEN^11^, PIK3CA 12, STK11^13^, and alterations in gene copy number, are associated with the pathological process of CSCC^14^, ^15^. In addition, the methylation of RMI2 and EPHX2 was closely linked to the clinical prognosis of CSCC^16^. It is still vital and pressing to uncover novel molecular mechanisms of cell proliferation, invasion *etc.,* in CSCC to develop valuable biomarkers or therapeutic targets for cancer identification and management.

Endothelial-specific molecule 1 (ESM1), also known as endocan, is a proteoglycan abundantly expressed in the vascular endothelium^17^. ESM1 has been implicated in the progression or metastasis of many malignancies, including pathogenic processes such as cell migration, invasion, and carcinoma angiogenesis^18^. It may serve as a potential biomarker for numerous cancers^19^. ESM1 expression was reported to be aberrant in several cancers, including triple-negative breast cancer^20^, hepatocellular carcinoma^21^, prostate cancer^22^, head and neck squamous cell carcinoma^23^, and others. Yet, the expression pattern, prognostic value, and biological mechanism of ESM1 in CSCC have mainly gone undetected.

In this paper, based on the IHC detection of ESM1 protein expression in clinical samples, related pathological parameters, and prognostic information, and combined with the assay of public tumor database, including TCGA and TNMpolter databases, and *in vitro* assays, we initially revealed that the expression of ESM1 was upregulated in CSCC, related to a dismal clinical outcome, and involved in signaling pathways related to cell proliferation and carcinoma angiogenesis. The findings in this work widen the knowledge of the role of ESM1 in the pathogenesis and development of CSCC and furnish vital insights for novel prognostic biomarkers or anticancer therapeutic targets for CSCC.

## Material and Methods

### Clinical samples

In this study, all formalin-fixed and paraffin-embedded (PPFE) specimens, including 145 CSCC and 45 paraneoplastic tissues, were gathered from February 2020 to February 2022 at our institution and then diagnosed and graded by over two pathologists and assessed for pathological features. Clinical sample inclusion criteria: (1) Pathologists diagnosed all tumor tissues derived from patients as cervical squamous cell carcinoma. (2) All relevant patients did not suffer preoperative radiotherapy or chemotherapy. (3) The patient had no other diseases or medical history except cervical cancer during the surgery. All the relevant clinicopathological signatures are shown in** Table 1**. Informed consent (IFC) was obtained from patients or their families for the samples involved in this study. This study was supported and approved by the Ethics Committee of Taihe Hospital (Ethical approval number, TH-2022-BLK-012-1).

### Immunohistochemistry (IHC) assay

We performed the IHC assay of all tissues with primary antibody (**Table S1**) followed by the protocol as previously described^24^. All IHC staining results were scanned and scored by at least two experienced pathologists. The ESM1 protein-positive cells were captured under a high-power (200×) microscopy field. The IHC assay of ESM1-positive cells was graded as follows: 0 (absent), 1 (<10%, weak staining), 2 (10%~50%, moderate staining) or 3 (≥50%, strong staining). All cases were classified into ESM1-high expression subgroup (ESM1^high^, score = 2 or 3) and ESM1-low expression subgroup ((ESM1^low^, score = 0 or 1).

### Bioinformatics identification

The public oncology database, including TCGA and TNMplot datasets, was gained for further bioinformatics identification. The TCGA-GTEx (The Cancer Genome Atlas Program and the Genotype-Tissue Expression Project) cohort contains 22 normal cervical tissues and 253 CSCC ones. The TNMplot (Differential gene expression analysis in Tumor, Normal, and Metastatic tissues) dataset^25^, displaying detailed analysis for a selected gene in a selected tissue type using gene-chip or RNA-Seq based data, provides 56 normal tissues and 189 CSCC ones.

### Clinicopathologic correlation analysis

The correlation between ESM1 expression and the clinicopathological characteristics of CSCC was investigated based on the follow-up data of our clinical samples. All clinical specimens were divided into two subgroups, including the ESM1^high^ group (n=46) and ESM1 ^low^ group (n=99). The Sanguini diagram was used to display the distribution of ESM1 expression in CSCC in terms of age, pathological TNM stage (pTNM), vascular invasion, lymph node metastasis and survival status^26^. Then, the cumulative survival of ESM1 expression in CSCC was analyzed based on follow-up information. We applied the univariate cox (uni-cox) and multi-cox (multi-cox) regression analyses to describe the effect of ESM1 expression on survival time and other clinical characteristics. Moreover, the clinicopathologic correlation analysis of ESM1 in CSCC was further verified in the TCGA database. The method details of the validation cohort of the TCGA dataset are included in** Supplementary Methods**.

### Gene-set enrichment analysis (GSEA)

The GSEA software 4.2.2 (Broad Institute, USA) was employed to characterize the potential biological mechanisms of ESM1 in TCGA-CSCC with samples from 31 patients in the ESM1 low expression subgroup (top 25%) and 30 patients in the ESM1 high expression subgroup (top 25%). Three predefined gene sets were analyzed, including 'h.all.v7.2.symbols.gmt', 'c2.cp.biocarta.v7.2.symbols.gmt' and 'c2.cp.go.v7.2.symbols.gmt'. Normalized Enrichment Score (NES) is the primary statistical result of GSEA with significance threshold: |NES| > 1 and normalized *P*-value < 0.05. Besides, the protein-protein interaction (PPI) networks were analyzed using the STRING database (version 11.5) to investigate the interactions between ESM1 and other genes.

### siRNA interference assay

The* in vitro* assay of siRNA knockdown in human CSCC cell lines, SiHa and ME-180 cells, was performed to explore the hidden molecular mechanisms of ESM1 in CSCC. More method details of the siRNA assay, Western blotting, cell proliferation assay, cell migration assay, cell invasion assay, and cell apoptosis analyses are included in **Supplementary Methods**.

### Statistical analysis

All statistics were undertaken by GraphPad Prism 8.0 software and SPSS 25.0. One-Way ANOVA or two-tailed Student's t-test, χ^2^-test, log-rank test and Pearson's correlation test were used in this study and indicated in the corresponding figure legends. The threshold for statistical significance was set at *P*<0.05.

## Results

### ESM1 expression is upregulated in CSCC compared to control cervical tissues

**Figure 1:**
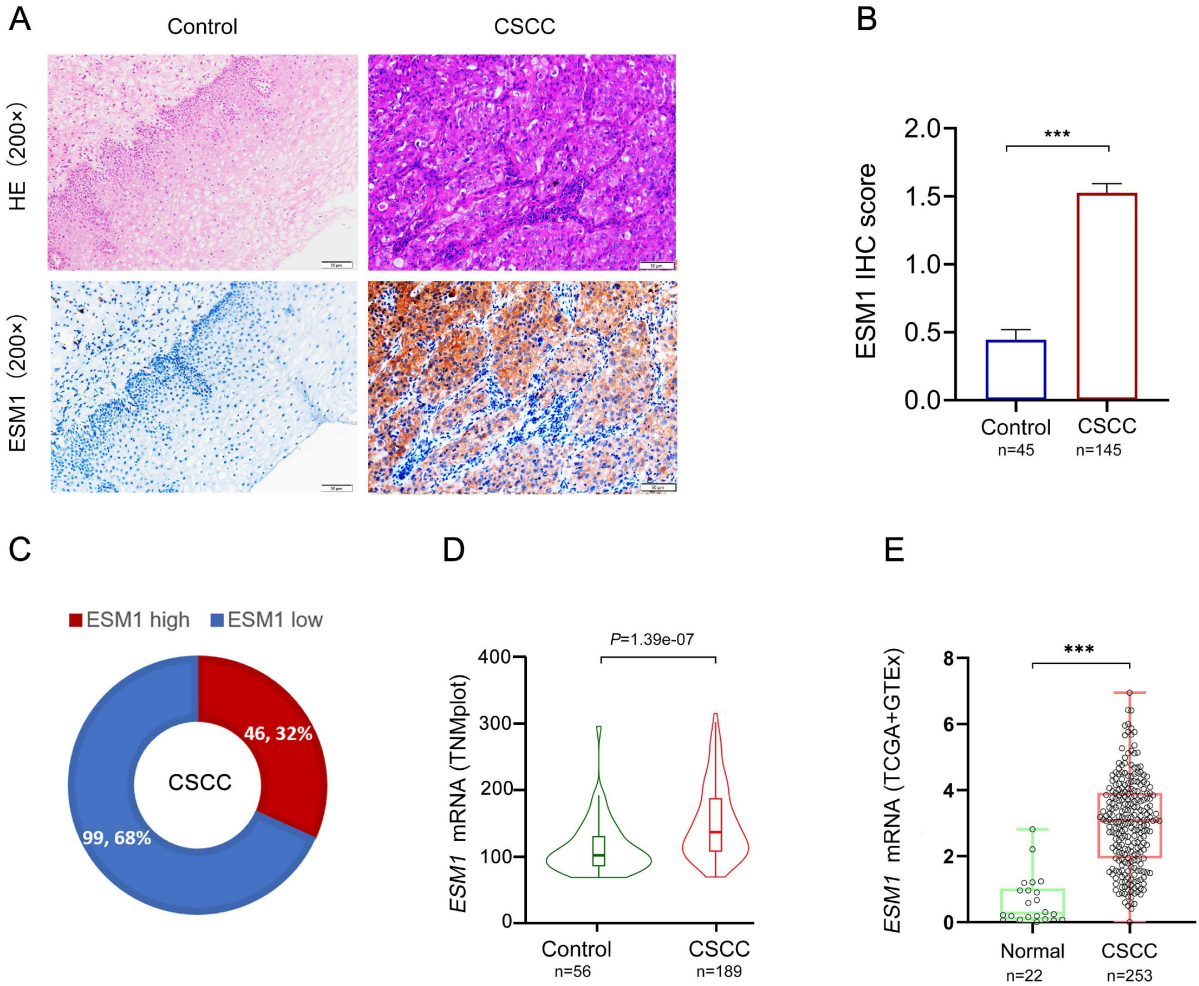
** The IHC staining of ESM1 in CSCC and control cervical tissues.** (A) The protein expression of ESM1 in cervical squamous cell carcinoma (CSCC) and paraneoplastic tissues (normal control) via immunohistochemistry assay (IHC; high power fields, 200×; scale bar, 50 μm). (B) The IHC score of ESM1 in CSCC (n=145) and control tissues (n=45). Data are shown as mean ± standard deviation (Mean ± SD). (C) The percentage of the ESM1 high and ESM1 low expression cases in all CSCC samples according to the IHC score. The relative mRNA expression of ESM1 from TNMplot platform (D) and TCGA-GTEx (The Cancer Genome Atlas Program and the Genotype-Tissue Expression Project) datasets (E). * *P*<0.05, ** *P*<0.01, *** *P* <0.001(Student's t test).

ESM1 expression was observed in human specimens via IHC assay, including control cervical tissue and CSCC samples. The findings revealed that the ESM1 protein was either not expressed or expressed at low levels in normal cervical tissues, yet hyper-expressed in CSCC. ESM1 protein was widely expressed in CSCC and was found mainly in the cytoplasm and intercellular matrix (**Figure 1A**). IHC scores showed that ESM1 was significantly overexpressed in CSCC compared to control cervical tissue (**Figure 1B**). All CSCC tissues (n=145) were divided into two groups, including 99 ESM1^low^ samples (score=0 and 1) and 46 ESM1^high^ samples (score=2 and 3). Consequently, the ESM1^high^ case was found in 32% (46/145) of the CSCC specimens **(Figure 1C)**. Based on the TNMplot and the TCGA datasets, bioinformatic analysis confirmed that ESM1 mRNA expression was hyper-expressed in CSCC compared to normal ones (**Figures 1D-E**).

### The association study of ESM1 and clinicopathological signatures in CSCC patients

**Figure 2:**
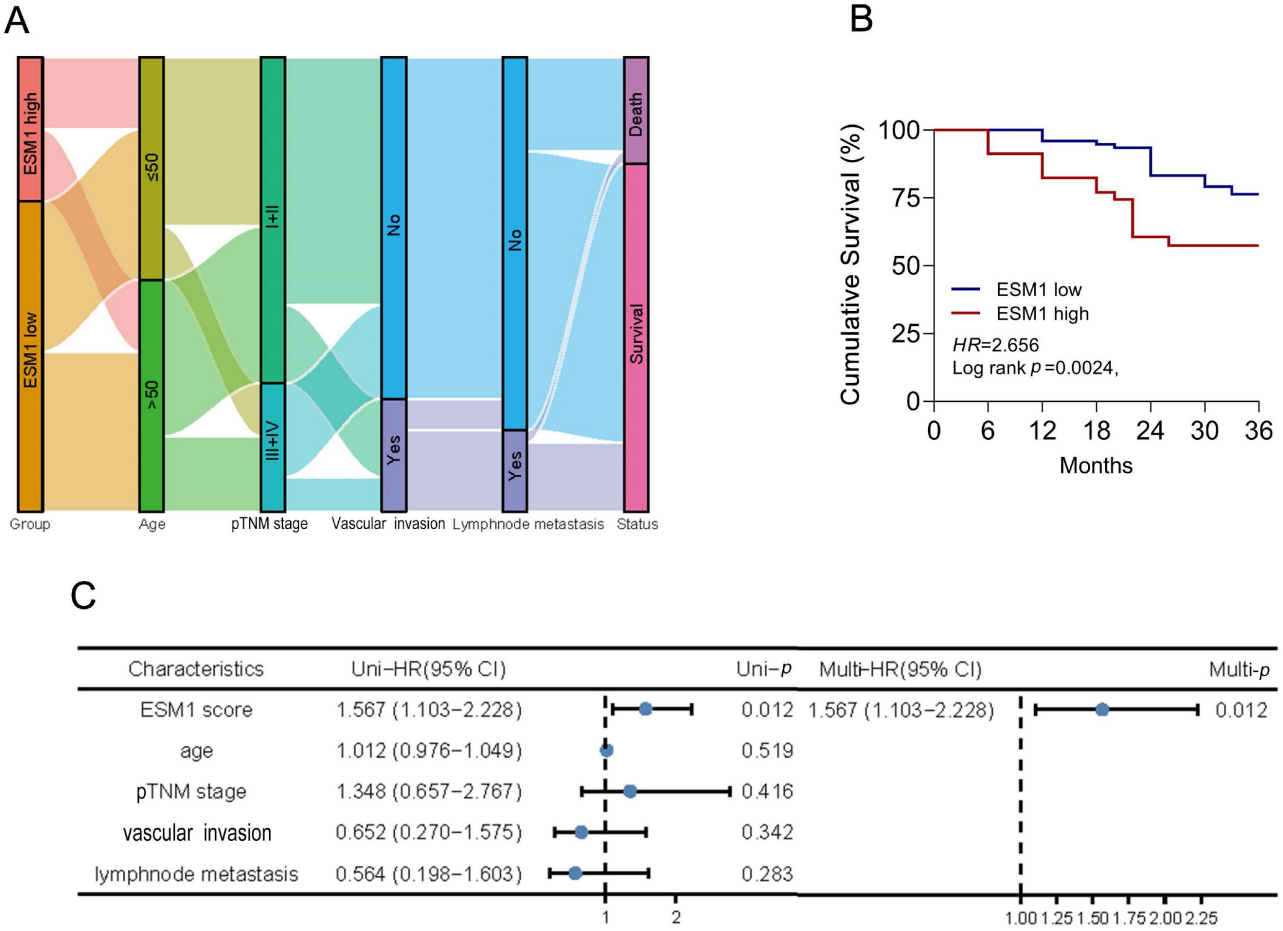
** Correlation and prognostic analysis of ESM1 expression in clinical pathological variables of CSCC patients.** (A) Sanguini plot for the analysis of the distribution of ESM1 expression in age, pTNM stage, vascular invasion, lymphnode metastasis, and survival status. (B) Kaplan-Meier curves of the cumulative survival for CSCC patients with ESM1 high and ESM1 low expression according to the IHC score. The log-rank test was applied for prognostic analysis. (C) The uni-cox and multi-cox analysis of ESM1 and relevant characteristics in CSCC. HR: hazard ratio; CI: Confidence interval.

The correlation study of ESM1 expression and clinicopathological characteristics in CSCC revealed that ESM1 expression was substantially associated with survival, pTNM stage, and vascular invasion but not with age or lymphnode dissemination (**Table 1**). CSCC patients with ESM1^high^ suffered worse survival status than those with ESM1^low^. It suggested that ESM1 has prognostic value and that overexpressed ESM1 may be unfavorable to CSCC patients. As shown in Figure 2A, the Sanguini diagram depicted the distribution of ESM1 expression concerning age, pTNM stage, vascular invasion, lymph node metastases, and survival status. Then, the prognostic analysis revealed that overexpressed ESM1 predicted poor cumulative survival in CSCC (Figure 2B). Moreover, the uni-cox analysis indicated that the ESM1 expression was closely associated with cumulative survival in CSCC patients; the multi-cox analysis asserted that ESM1 expression might be an independent prognostic parameter in CSCC (**Figure 2C**).

**Figure 3:**
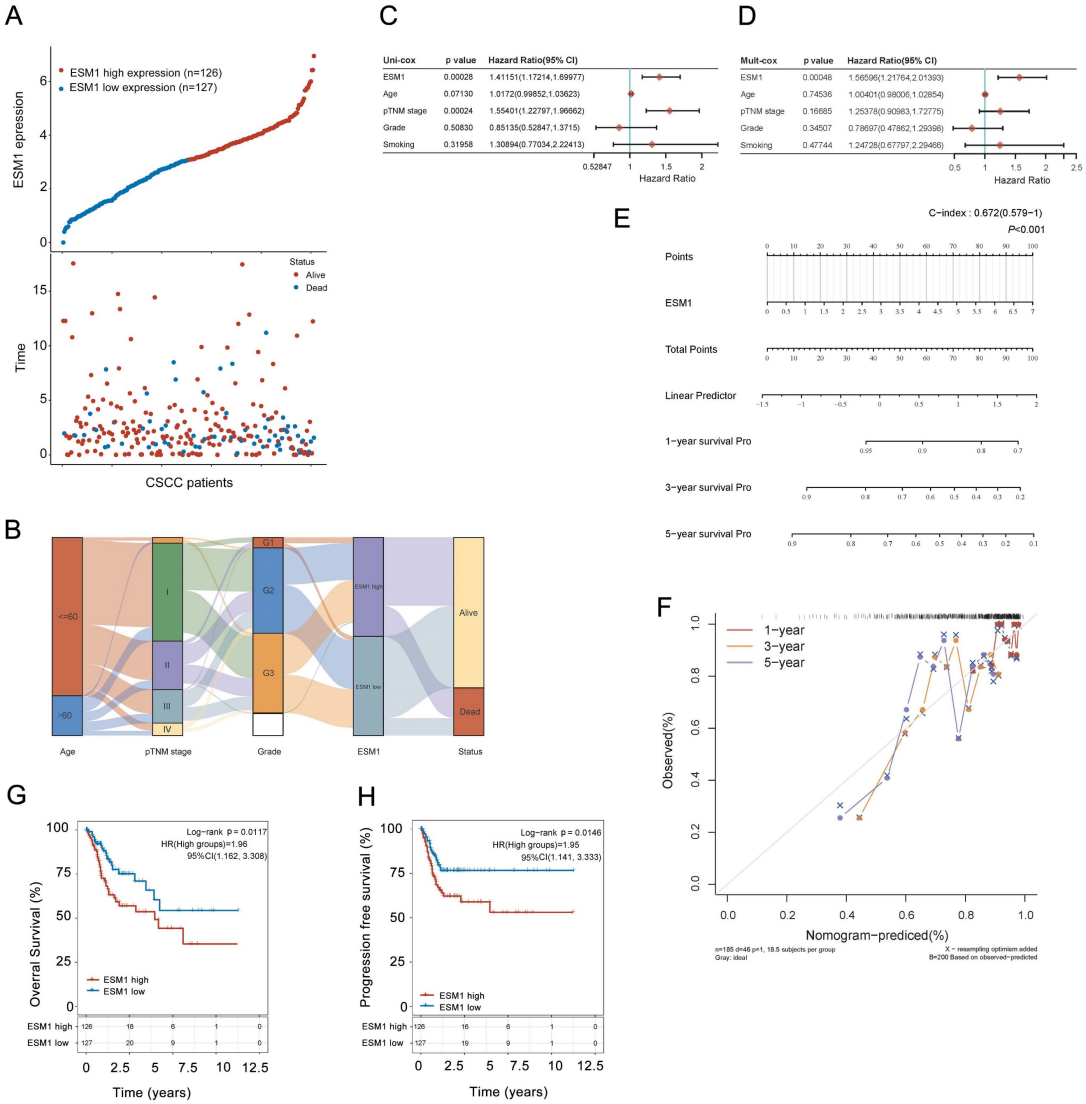
** Correlation and prognostic analysis of ESM1 with clinicopathological variables in the TCGA-CSCC cohort.** (A) Visualization of ESM1 expression (low or high) and survival status (dead or alive) in CSCC patients. The order of all samples was consistent. (B) The distribution of ESM1 expression in age, pTNM stage, grade and survival status is visualized by the Sanguini diagram. The uni-cox (C) and multi-cox (D) assay of ESM1 and relevant characteristics in CSCC patients. (E) Nomogram model for evaluating 1-, 3-, and 5-year survival of CSCC patients associated with ESM1 expression subgroup. (F) Calibration curves of the Nomogram model for the prognostic analysis of CSCC patients. The diagonal dashed lines indicate the ideal Nomogram model, and the blue, red, and orange lines indicate the predicted 1-year, 3-year, and 5-year Nomogram composite scores based on clinical parameters. The diagonal dashed line denotes the ideal Nomogram, and the blue, red and orange lines denote the 1-year, 3-year and 5-year observed Nomograms. The closer the Nomogram model is to the calibration curve, the better the model predicts the results. The prognosis analysis of ESM1 expression in CSCC patients, including the overall survival (OS; G) and progression-free survival (PFS; H) in the TCGA cohort. HR: hazard ratio; CI: Confidence interval.

In the validation cohort of the TCGA dataset, all TCGA-CSCC cases were divided into two subgroups, including 127 ESM1^low^ samples and 126 ESM1^high^ samples, to characterize the correlation between ESM1 and clinicopathological variables in CSCC (**Figure 3A**). Then, the Sanguini charts depicted the distribution of *ESM1* expression in different clinicopathological parameters, including age, grades, pTNM stage, and survival time (**Figure 3B**). It showed that ESM1 expression was substantially associated with survival status, but not with age, pathologic stage, or clinical grade (Table 2). The uni-cox and multi-cox regression analysis also stated that *ESM1* expression was closely correlated with survival status in CSCC patients (**Figure 3C**) and could be an independent prognostic factor for CSCC patients (**Figure 3D**). In addition, the Nomogram was used to predict survival at 1, 2, or 3 years in CSCC patients with high ESM1 expression. (**Figure 3E-F**). Finally, overexpressed ESM1 indicates poor OS (**Figure 3G**) and PFS (**Figure 3H**) in CSCC.

### GSEA analysis of ESM1 in CSCC

**Figure 4:**
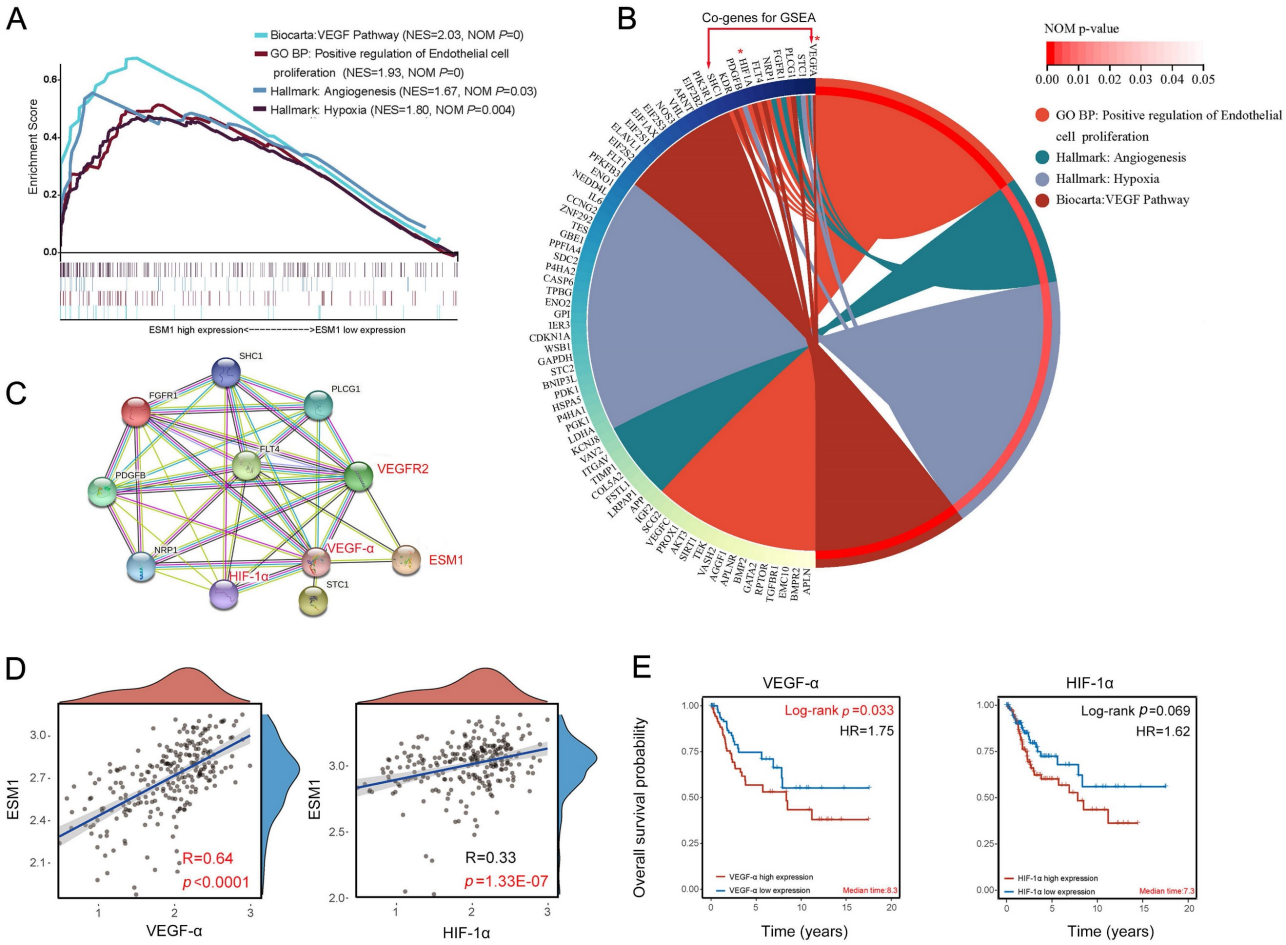
** Signaling pathways and co-expressed genes associated with ESM1 expression** (A) GSEA enrichment analysis: signaling pathways associated with high ESM1 expression in CSCC. (B) Circos's graph of co-expressed genes associated with the ESM1 signaling pathway. (C) PPI network of co-expressed genes. (D) Correlation of VEGFα, HIF-1α, and ESM1 expression in CSCC by Pearson's correlation test. (E) The OS analysis of VEGFα and HIF-1α expression in TCGA-CSCC.

The GSEA analysis was used to analyze the molecular mechanisms associated with ESM1 expression in CSCC. The GSEA enrichment analysis of the Biocarta pathway revealed that CSCC patients with high ESM1 expression were mainly associated with the VEGF pathway (**Figure 4A; Table S3**). Then, the GSEA analysis of GO terms suggested that the overexpression of *ESM1* was primarily enriched in the positive regulation of endothelial cell proliferation (**Figure 4A; Table S4**). The GSEA analysis of the Hallmark description uncovered that angiogenesis and hypoxia-related signaling pathways are mainly present in CSCC patients with high ESM1 expression (**Figure 4A; Table S5**). Circos' graph showed that VEGFα and HIF-1α were co-expressed and served crucial functions in these four signaling pathways (**Figure 4B**). The PPI network derived from the STRING database revealed a robust interaction between ESM1, VEGFα, HIF-1α and VEGFR2/3 (**Figure 4C**). Additionally, VEGFα and HIF-1α showed a positive correlation with the expression of ESM1(**Figure 4D**). Besides, overexpressed VEGFαand HIF-1α were associated with adverse OS of CSCC patients (**Figure 3E**). It indicates that ESM1 may have a critical regulatory role in modulating tumor angiogenesis and progression in CSCC by interacting with VEGFα and HIF-1α.

### ESM1 knockdown influences cell proliferation, invasion, and apoptosis in CSCC cell lines

**Figure 5:**
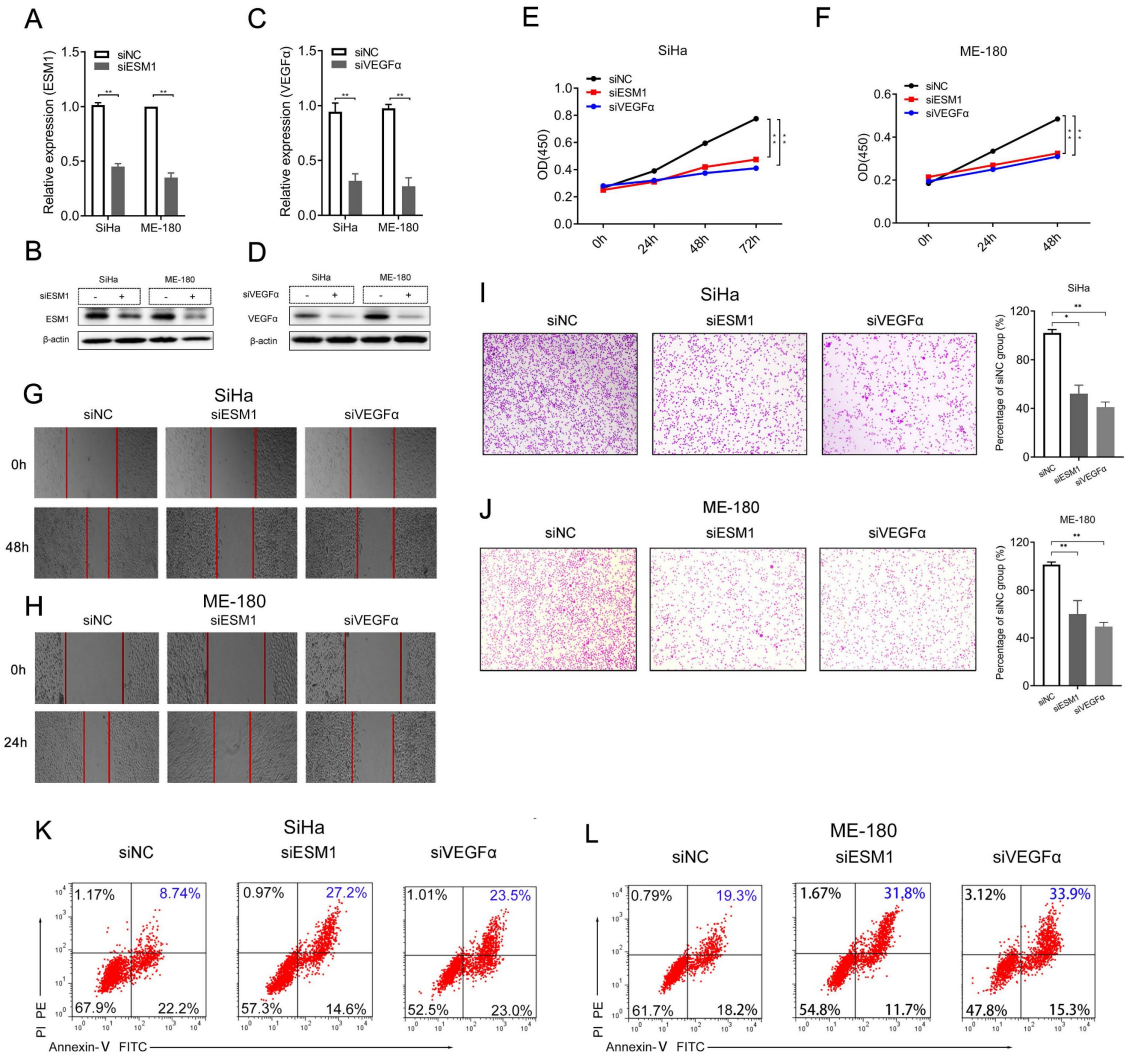
** Knockdown of ESM1 affects cell proliferation, migration, and apoptosis of SiHa and ME-180 cells.** (A-D) ESM1 mRNA and protein expression assessed by (A, C) RT-qPCR and (B, D) western blotting in SiHa and ME-180 cells after transfected with siRNAs. (E-J) Cell proliferation experiment (E, F), Cell mobility assay (G, H), cell invasion assay (I, J), cell apoptosis assay (K, L) of SiHa or ME-180 cells transfected with siESM1, siVEGFα, or siNC for 72 h and 48 h, respectively. * *P*<0.05, ** *P* <0.01, *** *P* <0.001 (Mean ± SD, Student's t-test.).

Then, we performed in vitro experiments with ESM1 siRNA (siESM1) in human CSCC cell lines, including SiHa and ME-180 cells, to further probe the role of *ESM1* in CSCC. The expression of mRNA and protein of siESM1 was significantly restrained compared to siNC. The ESM1 expression of mRNA and protein was significantly inhibited in siESM1 compared to siNC (**Figure 5A-B**). Specifically, interference with ESM1 significantly diminished cancer cell proliferation (**Figure 5E-F**), cell migration (**Figure 5G-H**), and cell invasion (**Figure 5I-J**) in human SiHa and ME-180 cells after being transfected with siRNAs for 48 h or 24h. Flow cytometry experiments revealed that downregulation of ESM1 dramatically increased cell apoptosis in SiHa (8.74% vs. 27.2%, **Figure 5K**) and ME-180 cells (19.3% vs. 31.8%,** Figure 5L**).

As presented in Figure 4, the expression of ESM1 was closely related to VEGFα in CSCC; siVEGFα was also designed to explore the correlation between ESM1 and VEGFα (**Figure 5C-D**). VEGFα knockdown significantly reduced cell proliferation (**Figure 5E-F**), cell mobility (**Figure 5G-H**), and cell invasion (**Figure 5I-J**) in SiHa and ME-180 cells after being transfected with siRNAs. Flow cytometry assay suggested that VEGFα interference significantly promoted cell apoptosis in SiHa (8.74% vs. 23.5%, **Figure 5K**) and ME-180 cells (19.3% vs. 33.9%, **Figure 5L**).

### ESM1 augments HIF-1α expression and the VEGFα signaling pathway in CSCC cells

**Figure 6:**
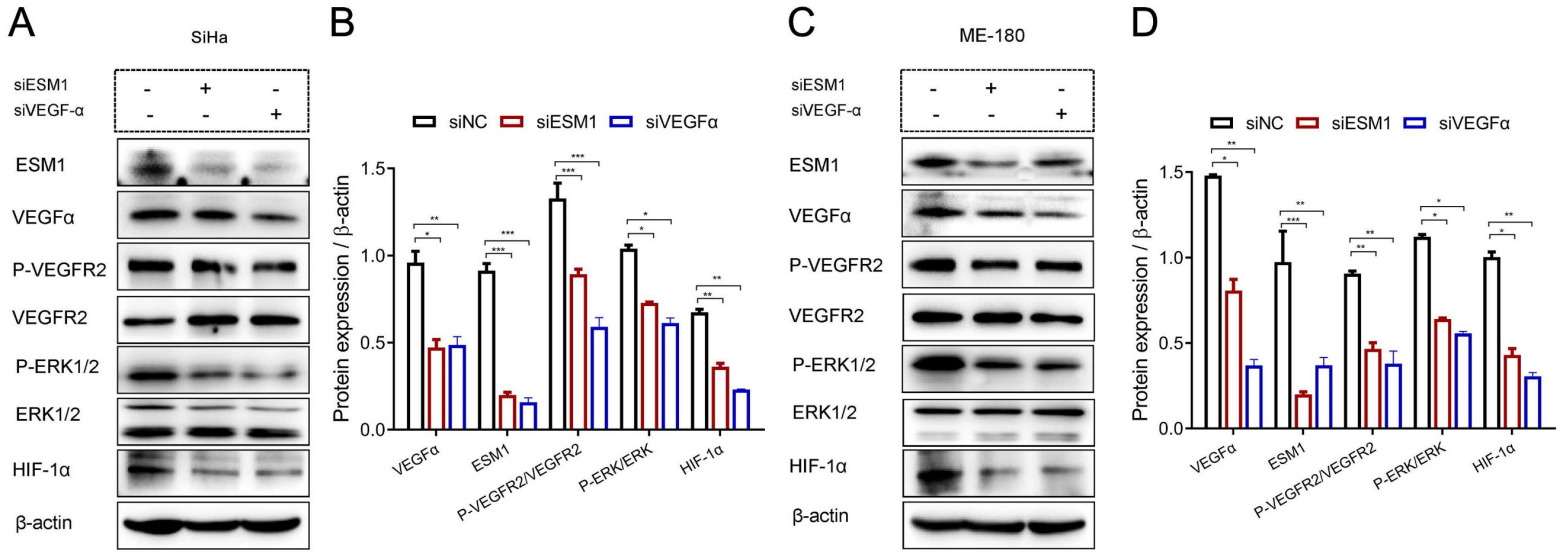
Western blotting assay of related signaling pathways after interference with ESM1 and VEGFα expression in SiHa (A) and ME-180 (B) cells, respectively. siRNA, small interfering RNA; NC, negative control; VEGFα, vascular endothelial growth factor α; HIF-1α, hypoxia-inducible factor 1 alpha; ERK, extracellular regulated protein kinase. * *P*<0.05, ** *P*<0.01, *** *P* <0.001 (Mean ± SD, Student's t-test.).

As shown in Figure 6, ESM1 knockdown decreased the expression of VEGFα and HIF-1α expression, and the phosphorylation of VEGFR2 (P-VEGFR2) and ERK-1/2 (P-ERK-1/2). Similarly, VEGFα inhibition dramatically decreased ESM1, HIF-1α expression, and phosphorylation of VEGFR2 (P-VEGFR2) and ERK-1/2 (P-ERK-1/2) in SiHa (**Figure 6A-B**) and ME-180 (**Figure 6C-D**) cells.

## Discussion

As one of the primary pathological histological subtypes of cervical cancer, CSCC is characterized by high malignancy, increasing prevalence yearly, and increasingly less mature age of onset^27^, ^28^. The primary therapeutic method for early-stage CSCC is surgical excision; patients at an advanced stage and losing the possibility of surgery need radiotherapy and chemotherapy, but the outcome is still unsatisfactory^5^. Thus, the search for early diagnostic and prognostic biomarkers or new therapeutic targets for patients with cervical cancer or non-HPV-associated cervical cancer failing to gain the HPV vaccine within the appropriate age range is meaningful to promote the clinical outcomes of CSCC.

As a 50 kDa soluble proteoglycan, ESM1 is primarily secreted by vascular endothelial cells. It is mainly expressed in vascular endothelial cells, distal renal tubular epithelial cells and lung endothelial cells of normal tissues, involved mainly in inflammatory responses^29^ and angiogenesis^30^. Evidence indicated that ESM1 was overexpressed in numerous malignancies and involved in neoplasm prognosis 31. In hepatocellular carcinoma, ESM1 enhanced tumor angiogenesis, especially in early tumor stages^21^. In triple-negative breast cancer, high expression of ESM1 promotes tumor cell migration, proliferation and invasion through the Akt/NF-κB/Cyclin D1 signaling pathway^20^. In prostate cancer, ESM1 maintains tumor cell stemness and metastasis by activating the Wnt/β-Catenin signaling pathway. Overexpression of ESM1 is associated with poor OS and distal metastasis in prostate cancer^22^. In esophageal cancer, ESM1 is an independent prognostic factor and facilitates the proliferation and migration of esophageal cancer cells through the Janus kinase (JAK) signaling pathway. Silencing the expression of ESM1 would overtly curb the cell proliferation and migration of esophageal cancer cells and decrease the expression of the JAK1 protein^19^. In head and neck squamous cell carcinoma (HNSC), ESM1 was significantly overexpressed, and it may promote HNSC progression through the Ras-MAPK-ERK signaling pathway^23^. Besides, ESM1 mediated the progression of non-small cell lung cancer with EGFR mutation^32^. However, the altered expression, clinical relevance, and biological functions of ESM1 in CSCC are still poorly known. The goal of this paper is to identify the expression pattern and masked clinical value of ESM1 in CSCC.

The IHC assay showed that ESM1 was not expressed or expressed at low levels in control cervical tissues while significantly overexpressed in CSCC. ESM1 protein was widely distributed in the cytoplasm and intercellular stroma of CSCC. In addition, the percentage of ESM1 highly expressed samples in CSCC was 32% (46/145). The hyper-expression of ESM1 was significantly related to survival status, pTNM stage, and vascular invasion. The uni-cox and multi-cox analysis hinted that ESM1 might be an independent prognostic factor and overexpression of ESM1 might be unfavorable to CSCC patients. Also, bioinformatics analysis of CSCC-related datasets (TNMplot and TCGA) further validated the above findings. Consequently, we confirmed through comparison that there is good consistency between our clinical sample dataset and validation set: ESM1 expression was upregulated in tumor samples and significantly correlated with poor prognosis of CSCC patients.

The GSEA analysis showed that ESM1 overexpression was associated with the co-expression of VEGFα and HIF-1α and was closely related to the VEGFα signaling pathway; ESM1 may regulate CSCC micro-angiogenesis and tumor progression via interaction with VEGFα and HIF-1α. Further, the siRNA assay of human CSCC cell lines, including SiHa and ME-180 cells, was structured to unlock the underlying molecular mechanisms. It reported that ESM1 knockdown suppressed the cell proliferation, migration, and invasion of SiHa and ME-180 cells and promoted cell apoptosis. Namely, the upregulation of ESM1 triggered cell proliferation, migration and invasion and arrested cell apoptosis. Conversely, interference of ESM1 leads to reduce the expression of VEGFα, HIF-1α, and the phosphorylation of VEGFR2 and ERK-1/2. Thus, the hyper-expression of ESM1 led to the upregulation of VEGFα and HIF-1α, and activated the VEGFα/VEGFR2/ERK signaling pathway. Additionally, silencing of VEGFα caused the block of ESM1, HIF-1α expression*,* and the phosphorylation of VEGFR2 and ERK-1/2. Consequently, it showed that ESM1 might regulate HIF-1α expression and activate the VEGFα signaling pathway in SiHa and ME-180 cells.

**Figure 7:**
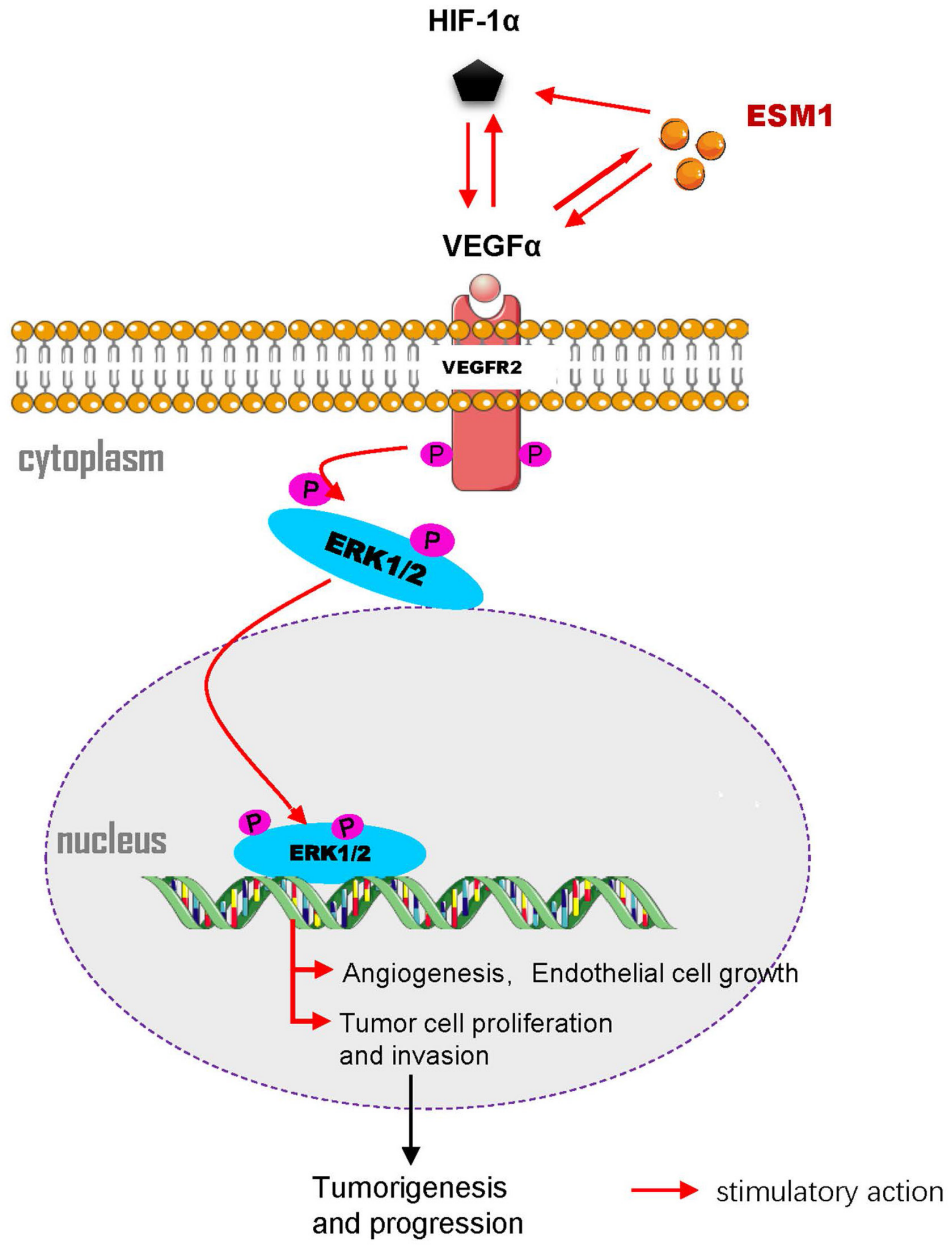
** Molecular signaling diagram.** Overexpressed ESM1 leads to induction of VEGFα and HIF-1α expression and activation of the VEGFα/VEGFR2/ERK signaling pathway, thereby promoting tumor angiogenesis, cell proliferation and invasion and arresting cell apoptosis.

Besides, previous reports are consistent with our results. HIF-1α modulated the expression of VEGFα, which caused angiogenesis and poor prognosis of various cancer^33^, ^34^. Malignant neoplasms are not only characterized by rapid proliferation but also form highly hypoxic zones and special micro-vessels in the core of the tumor^35^, ^36^. VEGFα and HIF-1α play crucial functions in tumor angiogenesis; HIF-1α can boost the mRNA stability of VEGFα and augment the transcriptional activity of VEGFα during hypoxia. Hence, VEGFα/HIF-1α is a promising biomarker for targeting tumor angiogenesis^37^. Based on the ESM1 knockout mouse model, upregulation of ESM1 expression promoted VEGFα expression and thus regulated endothelial cell proliferation^18^. In the assay of SiHa and ME-180 cells, ESM1 knockdown reduced VEGFα and HIF-1α expression. By contrast, VEGFα knockdown led to the reduction of ESM1 and HIF-1α expression. Therefore, this suggested that ESM1 and HIF-1α might have synergistic roles in tumor angiogenesis and cell proliferation, and they might promote CSCC progression through the VEGFα/VEGFR2/ERK signaling pathway (**Figure 7**).

The clinical significance and potential mechanisms of ESM1 or VEGFα had been individually and collectively investigated and elucidated in an amount of cancer, including cervical cancer, in previous studies. For instance, Li YK *et al.* showed that ESM1 overexpression facilitated cell proliferation, migration, and angiogenesis in ovarian cancer cells via the Akt pathway^38^. Furthermore, Lu JJ *et al.* revealed that ESM1 augmented the PI3K signaling pathway and accelerated EMT in cervical cancer^39^. Compared to these results, our research harbored the following differences: Firstly, we studied the clinical correlation between ESM1 and CSCC patients based on clinical sample studies and bioinformatics analysis of the TCGA database; The conclusions are consistent with previous studies; Then, through *in vitro* experiments, a preliminary study was conducted on the expression of ESM1 through VEGFα/VEGFR2/ERK and hypoxia-related signaling pathways regulate tumor microvascular formation; The different molecular mechanism compared to previous studies suggested that ESM1 might participate in the occurrence and development of CSCC through multiple avenues. Our study not only confirmed the relevant conclusions of other literature but also widened the molecular mechanism of ESM1 as a therapeutic target, providing clues for further* in vivo* study.

In this study, we explored the underlying biological features of overexpressed ESM1 in human CSCC via the VEGFα/ERK signaling pathway to augment endothelial cell proliferation and tumor angiogenesis, suggesting that ESM1 may serve as a promising prognostic biomarker or therapeutic target for CSCC. However, our study still suffers from some limitations. Firstly, the molecular mechanism of ESM1 in CSCC is only based on GSEA analysis and *in vitro* assays of human CSCC cell lines. Therefore, conducting in vivo study or further clinical research on ESM1 in CSCC patients is still necessary. Then, as a secreted protein, the expression of ESM1 in the blood of CSCC patients was not validated, so further discussion on the latent diagnostic or prognostic significance of ESM1 in the blood samples of CSCC patients is required. Accordingly, more *in vivo* studies and blood samples related to the research of CSCC patients are still needed to confirm the above findings.

## Conclusion

In conclusion, this work demonstrates that ESM1 is significantly overexpressed in CSCC patients, is an independent prognostic factor, and predicts a poor clinical outcome. ESM1 may be considered an indicator of prognosis or a therapeutic target of CSCC patients. Besides, ESM1 and HIF-1α harbored synergistic functions in tumor angiogenesis and cell proliferation of CSCC cells via the VEGFα/VEGFR2/ERK signaling pathway based on *in vitro* experiments. Briefly, this study furnished valuable evidence for the role of ESM1 and related signaling pathways in the occurrence and development of CSCC, which may give helpful insight for developing promising or individualized therapeutic strategies for CSCC patients with high ESM1 expression.

## Supplementary Material

Supplementary methods and tables.

## Figures and Tables

**Table 1:** Correlation between ESM1 expression and clinicopathological characteristics in CCSC patients (n=145).

Characteristic	ESM1 expression		χ^2^	*P-*value	Characteristic	ESM1 expression			χ^2^	*P-*value
	Low (n=99)	High (n=46)				Low (n=99)		High (n=46)		
**Age**			0.028	0.87	**Vascular invasion**				7.38	0.0066^a^
≤50	48	23			Yes	81		28		
>50	51	23			No	18		18		
**Status**			6.85	0.0089^a^	**Lymphnode metastasis**				0.12	0.73
Alive	82	29			Yes	82		37		
Dead	17	17			No	17		9		
**pTNM stage**			7.68	0.0056^a^						
Stage I+II	78	26								
Stage III+ IV	21	20								
										

Note: pTNM stage, the pathologic TNM stage, which was assessed according to the 9th edition of the American Joint Commission on Cancer (AJCC); a, χ^2^ test and *P*<0.05

**Table 2:** Correlation between ESM1 expression and clinicopathological characteristics in TCGA-CCSC patients.

Characteristic	ESM1 expression level		*P* value	Characteristic		ESM1 expression level			*P* value
	Low (n=126)	High (n=127)				Low (n=126)		High (n=127)	
**Age**			0.99	**Clinical grade**					0.128
Mean (SD)	48.8 (14.4)	47.2 (13.8)		Grade 1		6		7	
Median [min, max]	45.5 [21,88]	48 [20,81]		Grade 2		61		48	
**Status**			**0.021**	Grade 3	49		53		
Alive	104	88		Grade 4		10		19	
Dead	22	39							
**Pathologic stage**			0.109						
Stage I	67	54							
Stage II	30	32							
Stage III	18	22							
Stage IV	11	19							
									
