# Supplementary material for: Targeting ESM1/ VEGFα signaling axis: a promising therapeutic avenue for angiogenesis in cervical squamous cell carcinoma
Source: J Cancer. 2023 Jun 12;14(10):1725–35. doi: 10.7150/jca.84654 (PMC10355198; doi:10.7150/jca.84654)
Supplement: Supplementary file 1 — Supplementary methods and tables. [file jcav14p1725s1.zip › Supplementary material/Supplementary Methods.docx]

**Supplementary Methods:**

**1. Clinicopathological correlation from TCGA dataset**

As the validation cohort of the TCGA dataset, a total of 253 CSCC patients were assigned into two subgroups, including 126 specimens with *ESM1* high expression (ESM1^high^) and 127 specimens with *ESM1* low expression (ESM1^low^) by the median cutoffs. The 'ggrisk' package of R software (version 4.0.3) was applied to cluster the expression groups^[1]^. The distribution of ESM1 expression in ages, stages, grades, survival status, and other clinical characteristics for CSCC was displayed by the Sanguini diagram. In addition, the uni-cox and multi-cox regression analyses were performed. Several features, including p-values, hazard ratio (HR), and 95% confidence interval (CI), were presented by 'forest plot' R packages^[2]^. Lastly, the prognostic analysis of CSCC with ESM1^high^ and ESM1^low^ was conducted, including overall survival (OS) and progression-free survival (PFS).

**2. siRNA knockdown**

### 2.1 The siRNA assay and Western blotting

Human CSCC cell lines, including SiHa and ME-180 cells, were transfected with 50 nmol of ESM1 siRNA (siESM1), VEGFα siRNA (siVEGFα), or negative control siRNA (siNC) in the special medium (CM0451, Procell, China) for 48 h and 24 h, respectively, according to the manufacturer's instructions. Then, total RNA was isolated by TRIzol reagent (Invitrogen, USA) and RT-qPCR was performed as previously described^[3]^. The relative expression of ESM1 and VEGFα was calculated in three replicates. GAPDH was utilized as an endogenous reference gene. All reactions were run in triplicate. The relative *ESM1* mRNA expression was assessed by using the 2^−ΔΔCt^ method. All primers were manufactured by Sangon Biotech (Shanghai, China) and the corresponding sequences were presented in **Table S3**. Meanwhile, other samples under the same culture conditions were used to lyse the protein for western blotting.

### 2.2 Cell proliferation assay

A total of 100 μL (1×10^4^ cells) of human SiHa or ME-180 cells were inoculated in 96-well plates and transfected with 50 nmol of siESM1, siVEGFα or siNC, followed by the addition of 10 μL of CCK-8 solution (Beyotime, China) in each well. After 72 h or 48 h of incubation, the absorbance (optical density, OD) was measured at 450 nm, representing the density of cells.

### 2.3 Cell migration assay

Cell migration was analyzed by wound-healing assay. Human SiHa and ME-180 cells were plated in 12-well plates in DMEM containing 10% fetal bovine serum (FBS) and transfected with 50 nmol of siESM1, siVEGFα or siNC for 48 h and 24 h, respectively. The floating cells were washed with PBS and then 4 ml of DMEM (10% FBS, 1% antibiotic-antimycin) was added. Scratch areas were drawn at time endpoints.

### 2.4 Cell invasion assay

Human SiHa and ME-180 cells transfected with siESM1, siVEGFα, or siNC were cultured in TranswellR cell culture chamber (Corning, USA) at 1×10^4^ cells/well for 48 h and 24 h, respectively. After 24 h of culture, cells under the membrane were fixed with 4% PFA and stained with crystal violet. Cells in 5 random areas of each chamber were photographed and counted using Image J software.

### 2.5 Cell apoptosis analyses

After transfection of human SiHa and ME-180 cells with siESM1, siVEGFα or siNC for 48 h and 24 h, respectively, the cells were disposed of with Annexin V-FITC kit (Beyotime Biotechnology, China) and analyzed by flow cytometry (FACSCalibur, Bio-Rad, USA) for the analysis of apoptotic cells.

# References

1. Zhang Z, Lin E, Zhuang H, Xie L, Feng X, Liu J *et al*: Construction of a novel gene-based model for prognosis prediction of clear cell renal cell carcinoma. *Cancer Cell Int* 2020, 20:27-44.

2. Xiong Y, Yuan L, Xiong J, Xu H, Luo Y, Wang G *et al*: An outcome model for human bladder cancer: A comprehensive study based on weighted gene co-expression network analysis. *J Cell Mol Med* 2020, 24(3):2342-2355.

3. Huang Y-g, Li D, Wang L, Su X-m, Tang X-b: CENPF/CDK1 signaling pathway enhances the progression of adrenocortical carcinoma by regulating the G2/M-phase cell cycle. *Journal of Translational Medicine* 2022, 20(1):78-94.
