# Supplementary material for: Targeting ESM1/ VEGFα signaling axis: a promising therapeutic avenue for angiogenesis in cervical squamous cell carcinoma
Source: J Cancer. 2023 Jun 12;14(10):1725–35. doi: 10.7150/jca.84654 (PMC10355198; doi:10.7150/jca.84654)
Supplement: Supplementary file 1 — Supplementary methods and tables. [file jcav14p1725s1.zip › Supplementary material/Table S1 . Details of all antibodies.docx]

**Table S2. Details of all antibodies involved in this study.**

| **Antibodies** | **Manufacturer brand** | **Catalogs NO.** | **Dilute proportion** | **Molecular weight** |
| --- | --- | --- | --- | --- |
| ESM1 antibody | Bioss, USA | Bs-3615R | IHC,1:200; WB,1:500 | 20kDa |
| VEGF-α | Affinity biosciences, USA | AF5131 | IHC,1:200; WB,1:1,000 | 25kDa |
| HIF-1α | Affinity biosciences, USA | A1009 | IHC,1:200; WB,1:1,000 | 120kDa |
| VEGFR2 | Affinity biosciences, USA | AF6281 | WB,1:1,000 | 180kDa |
| p-VEGFR2 | Affinity biosciences, USA | AF3279 | WB,1:1,000 | ~170kDa |
| ERK1/2 | Affinity biosciences, USA | AF0155 | WB,1:1,000 | 42kDa |
| p-ERK1/2 | Affinity biosciences, USA | AF8208 | WB,1:1,000 | ~42kDa |
| CD31 | MXB, China | MAB-0720 | IHC,1:100 | 25kDa |
| β-actin antibody | Santa Cruz, USA | Cat:sc-47778 | WB,1:200 | 42kDa |
