# Supplementary material for: Targeting ESM1/ VEGFα signaling axis: a promising therapeutic avenue for angiogenesis in cervical squamous cell carcinoma
Source: J Cancer. 2023 Jun 12;14(10):1725–35. doi: 10.7150/jca.84654 (PMC10355198; doi:10.7150/jca.84654)
Supplement: Supplementary file 1 — Supplementary methods and tables. [file jcav14p1725s1.zip › Supplementary material/Table S2 . The sequences of primers and siRNAs..docx]

**Table S3.** The sequence information of RT-qPCR primers and siRNAs.

| **Gene name** | **Primers (5**'-**3**') | |
| --- | --- | --- |
| ***Hu-ESM1*** | Forward: CTTGCTACCGCACAGTCTCA | Reverse: GCCATGTCATGCTCTTTGCAG |
| ***Hu-VEGFα*** | Forward: TGGCTCACTGGCTTGCTCTA | Reverse: ATCCAACTGCACCGTCACAG |
| ***Hu-GAPDH*** | Forward: CGGAGTCAACGGATTTGGTCGTAT | Reverse: AGCCTTCTCCATGGTGGTGAAGAC |
| **si-ESM1** | Forward: ACUUGUAUGUGUUUGUUAAAU | Reverse: UUAACAAACACAUACAAGUGU |
| **si-VEGFα** | Forward: CCGAAACCAUGAACUUUCUTT | Reverse: AGAAAGUUCAUGGUUUCGGTT |
| **si-NC** | Forward: UUCUCCGAACGUGUCACGUTT | Reverse: ACGUGACACGUUCGGAGAATT |

si-ESM1: *ESM1* siRNA; si-VEGFα: VEGFα siRNA; si-NC: negative control siRNA, as scramble.
